# Ectropion and Entropion1Read at the Thirteenth Semi-Annual Meeting of the Illinois Veterinary Medical and Surgical Association, August 15, 1902.

**Published:** 1902-08

**Authors:** S. H. Swain

**Affiliations:** Decatur, Ill.


### ECTROPION AND ENTROPION.[1]

By S. H. Swain, V.S.,
DECATUR, ILL.

Eversion of the eyelids may be acute and temporary, or may be the consequence of chronic alterations or of traumatic injuries of contiguous parts, or a result of defect in the facial nerves supplying the orbicular muscles, or it may be due to acute inflammation causing hypertrophy of the conjunctival membrane; the eyelid is thus drawn away from the eyeball, its conjunctival surface turned out, and its cilia margin misplaced downward or upward according as the inferior or superior lid is the seat of the ectropion. The eyeball being thus deprived of the protection of the eyelid is exposed to continuous irritation, resulting in chronic conjunctivitis, weakening the eye, and finally causing partial or complete amaurosis.

The canine seems more subject to this malady than the equine or other families of domestic animals, and the inferior eyelid more subject to attacks than the superior lid.

[1] Read at the Thirteenth Semi-Annual Meeting of the Illinois Veterinary Medical and Surgical Association, August 15, 1902.

TREATMENT. If the ectropion is the result of an attack of acute inflammation it may then be successfully treated by frequent applications of mild astringent collyria, and may be composed of zinc sulphate, 2 grains; morphine sulphate, ½ grain, to 1 ounce of distilled water; or the following cooling lotion may be prescribed: boric acid, 10 grains to 1 ounce of distilled water, frequently and liberally applied; but if this astringent treatment should after a fair trial fail to effect a cure the eyelid may then be further everted and carefully curetted and then pencilled with nitrate of silver from one angle of the eye to the other, parallel to but at a little distance from the margin of the eyelid; after the lapse of a few moments the eye may be carefully washed out with clean water and treated as before mentioned with cooling astringent lotions, and if necessary the pencilling with nitrate of silver may be repeated in about ten days, and in the event of failure from the above treatment the surgeon should at once resort to an operation. The eyelid should be drawn away from the eyeball, the hypertrophied conjunctiva pinched up and elevated by means of suitable forceps, and a section of sufficient length and breadth removed to correct the ectropion, which will require on the part of the operator accurate judgment, skill, and dexterity; for if the section removed should not be sufficiently large a slight ectropion will still remain after the wound has cicatrized; then, again, if the section removed should be too large the result will be entropion after cicatrization has been completed. The after-treatment should consist of cleanliness and mild astringent lotions frequently applied as above mentioned, with light, easily digestible food, diuretics, and physics if indicated.

### *Entropion.*

This is the converse position of the eyelids to that of ectropion. The free margin of the eyelids and the cilia are turned in against the eyeball, keeping up a constant state of irritation, and the pain and distress caused by the friction of the margin of the eyelid and the eyelashes must be very excruciating to the poor sufferer.

The time was when all these conditions of the eye were attributed to wild or abnormally developed eyelashes, and the empirics could always be found with tweezers in hand extracting wild eyelashes for the imaginary cure of any and all affections of the eye; now, it is well understood by the profession that these so-called wild hairs are only inverted eyelashes, a condition known as trichiasis, and may be successfully treated by first extracting the offending hair and then piercing the seat of the hair—the hair follicles—with an electric cautery needle.

The symptoms are intolerance to light, the animal keeping the affected eye closed almost all the time; there is increased lacrymation, with the general symptoms of conjunctivitis. Entropion may be congenital, partial, or complete, or it may occur at any period of life from loss of tone in the levator palpebræ muscles, or it may result from rupture and collapse of the eyeball, causing the eyeball to lose its spheroidal shape, thereby permitting the eyelids to be inverted upon themselves, constituting entropium.

Treatment consists in excision of an oval or elliptical section of the eyelid sufficient to correct the entropion. The operation should be performed as near the margin of the eyelid as possible, taking care to leave sufficient breadth of skin at the margin of the eyelid for the insertion of stitches. After the excision of the parts designated above, the edges of the wound are to be carefully brought together by metallic sutures and the wound dressed with dry, antiseptic powder; probably one of the best is composed of iodoform, one part; boric acid, three parts; mixed and dusted on once or twice daily. The horse or other animal should now be so secured as to effectually prevent chafing of the wound. The indications for feeding, watering, and the constitutional treatment are the same as advised in ectropion.

---

GLYCOSURIA OF MUSCULAR ORIGIN. Cadéac and Maignon have demonstrated: (1) That glycosuria may result from crushing of the muscles, by ligaturing a leg; and (2) that this glycosuria is of muscular origin. This has been observed in the dog and guinea-pig by ligaturing the thigh or crushing the muscles with pincers. Clinical observation has also demonstrated this in the dog in fractures and crushing of the legs from accidents. Phenylhydrazin demonstrated the presence of glucose or glycosuria in the urine two or three days after the accident, which persisted from three to four weeks; that is, until the local alterations had disappeared.

The filtrate obtained by macerating for an hour, in distilled water, normal and crushed muscle showed the presence of only a trace of sugar in the former and a marked quantity in the latter. It seems, therefore, that at times diabetic urine may be of muscular origin.—*Rec. de Med. Vet.*

